# Study on the Application of the Kent Index Method on the Risk Assessment of Disastrous Accidents in Subway Engineering

**DOI:** 10.1155/2013/360705

**Published:** 2013-04-09

**Authors:** Hao Lu, Mingyang Wang, Baohuai Yang, Xiaoli Rong

**Affiliations:** ^1^College of Defense Engineering, PLA University of Science and Technology, Nanjing 210007, China; ^2^Nanjing KunTuo Civil Engineering Technology Co., Ltd., Nanjing 210007, China

## Abstract

With the development of subway engineering, according to uncertain factors and serious accidents involved in the construction of subways, implementing risk assessment is necessary and may bring a number of benefits for construction safety. The Kent index method extensively used in pipeline construction is improved to make risk assessment much more practical for the risk assessment of disastrous accidents in subway engineering. In the improved method, the indexes are divided into four categories, namely, basic, design, construction, and consequence indexes. In this study, a risk assessment model containing four kinds of indexes is provided. Three kinds of risk occurrence modes are listed. The probability index model which considers the relativity of the indexes is established according to the risk occurrence modes. The model provides the risk assessment process through the fault tree method and has been applied in the risk assessment of Nanjing subway's river-crossing tunnel construction. Based on the assessment results, the builders were informed of what risks should be noticed and what they should do to avoid the risks. The need for further research is discussed. Overall, this method may provide a tool for the builders, and improve the safety of the construction.

## 1. Introduction

Creating a perfect design in subway engineering is difficult because of complex geological environment and difficulties in completely obtaining basic information. The influence of current large-scale subway construction, the limited construction period, and poor management caused by the lack of skilled personnel contribute to the increase in the occurrence of accidents in subway construction. Thus, the issue of safety has become very serious [1, 2]. Accidents indicate that subway construction affects the ambient environment (ground buildings, transportation, underground structures, underground pipes, etc.), endangers people's lives, compromises property security, and causes serious economic losses [2]. Several typical subway construction accidents are shown in Table 1. 

Plenty of new urgent tasks are being proposed because of the serious safety issue in subway engineering. One of these tasks is to study the safety risk management method. In recent years, the utilization of risk assessment in subway engineering has significantly increased and has provided particular economic benefits and research results [3–5].

The book “Code for Risk Management of Underground Works in Urban Rail Transit” [6] published in 2011 provides a reference for the application of risk management in subway engineering and considers the classification standard of probability and consequence. However, in the application process, the risk factors that influence scope, occurrence mechanism, and potential damage mechanism in subway construction are very complex. Risk management involves many disciplines such as natural science, social science, engineering technology, system science, and management science. Thus, determining if a probability distribution hypothesis is appropriate becomes difficult when tunnel and underground engineering risks are studied with the probability method [7, 8]. Thus, obtaining the “real” probability value of an accident is difficult [9]. 

Kent used the index method to study pipeline accidents. He believes that pipeline accidents cannot be accurately predicted, and risk assessment does not provide an accurate calculation based on the probability theory. Insufficient sample size or calculation quantity is usually regarded as the reason for the inaccurate calculation, but in truth, the main reason is that too many assumptions are made in the computation or collection of samples, which leads to the inaccuracy of the assessment result. Kent's method does not consider the “real” probability; the indexes in Kent's method contain the probability and are not tied to the “real” probability, which is very persuasive [9].

By adopting advanced techniques from the Kent index method and considering the limitation of the application of Kent's method in subway engineering, a model that can be applied to risk assessment of disastrous accidents in subway engineering is developed in this paper. This paper also provides a reference for quantitative evaluation of disastrous risks involved in subway engineering and other similar fields.

## 2. Improvement of the Kent Index Method

Kent's method does not intentionally evade the subjective factors in risk assessment. In fact, his method adopted several feasible measures to reduce the negative influence of these factors, thereby providing a good reference for risk assessment in subway engineering. Several researchers have questioned the expert scoring method because of its alleged subjectivity. In truth, opinion, experience, intuition, and other unquantifiable resources are used if knowledge on the matter is limited. Thus, risk assessment becomes at least partially subjective [9]. Moreover, subjectivity is found in any and all risk assessment methodologies. However, experts also have limitations. The assessment results obtained through the expert scoring method could be inconsistent because of the discrepancy between individual and diffused thought. In the same way that experts need the guidance of a risk manager to normalize their thoughts, several research methods (including theoretical research, value simulated, test demonstration, etc.) should be used to minimize subjective influences.

Kent's method has many advantages. Thus, it is extensively applied in pipeline risk assessment. Unfortunately, its disadvantages restrict its application in other fields.

One of the disadvantages of Kent's method is that no specific method or train of thought is used to determine weight, which is very important in risk assessment. Weight is mainly obtained through the experience of an expert, and no perfect solution exists to solve this problem. In the opinion of the author, determining the weight value is an iterative process whose result can be perfected through repeated application.

Another disadvantage is the assumption regarding the independence of the indexes. In risk analysis, the indexes are assumed to be independent. This assumption could have different effects on the analysis of different objects. Thus, the method is not suitable for all cases.

Pipeline risk is a relatively simple problem, only one accident, the bursting of the pipeline. In underground engineering, for example, the common risk factors include collapse, water gushing, surface subsidence, and so on, and a significant correlation exists among these factors. Thus, ensuring construction safety by merely applying Kent's method is difficult. The method must be modified before being applied in underground engineering safety risk assessment.

Several suggestions have been proposed for the application of Kent's method in underground engineering risk assessment.

- Practical engineering should be the basis of research; the basic concept of risk must be utilized to make risk assessment more feasible.
- In determining the weight value and other data, existing research results should be considered together with an expert scoring method.
- An effective assessment cannot be achieved by mere calculation of the summing indexes because of the complex relationship among strata condition, design, and construction (they affect one another). Thus, the model needs to be improved first.

## 3. Improved Index Method Model

### 3.1. Index Categories

Based on the characteristics of subway construction, indexes are divided into four categories, namely, basic, design, construction, and consequence indexes. The diversity of subway construction methods causes the indexes to be different in the different construction methods. In this paper, the shield method of construction is utilized as an example.

The basic indexes mainly refer to the attributes of engineering that cannot be routinely changed and are beyond the control of the operators. These attributes are determined immediately after line selection, which considers existing hydrological conditions [14, 15], geological conditions [16], surrounding environment conditions, and tunnel parameters including the size of the tunnel, slope, turning radius, and so on [17].

“Design” in this paper refers to the idea that can prevent the risks caused by the basic indexes and provide convenience and guidance in the construction process. It is the precondition of construction. In view of the characteristics of shield tunneling, the main design indexes include the following: reinforced design, environmental protection design, precipitation design, construction method design, shield machine design, and segment design [18].

The construction indexes mostly focus on management and operation during the period of construction. They mainly include the selection of construction methods and level of construction technology, analysis of the influence of the construction period, specification of the external construction environment, and control measures.

The consequence indexes include five aspects, namely, environmental loss, economic loss, social loss, casualties, and construction time loss.

**Figure 1:**
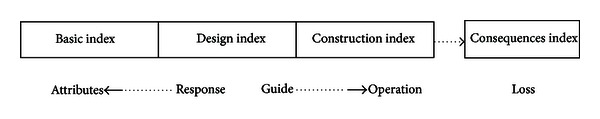
Relationship between indexes.

The definition and relationship of these four kinds of indexes are shown in Figure 1.

### 3.2. Risk Assessment Model

The most commonly accepted definition of risk is often expressed as a mathematical equation:

(1) R=f(P,c),

where *R*: risk; *P*: event probability; *c*: event consequence.

 Considering the basic model, the index method model can be expressed as

(2) R=P×c,P=f1(B,D,C),c=f2(consequences index),

where *B*: basic index; *D*: design index; *C*: construction index.

In the above expression, *P* does not represent the exact probability, only the probability index. The scope of *P* is not from 0 to 1; however, its value contains the meaning of probability and has a positive correlation with the real probability (the higher the value, the greater the probability of risk).

**Figure 2:**
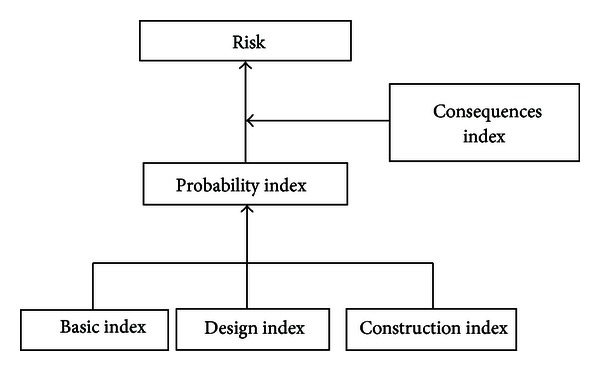
Improved index method model.

The probability index is composed of the basic, design, and construction indexes. The consequence index represents losses caused by an accident or accidents, as shown in Figure 2.

### 3.3. Probability Index Model

The probability index is an important part of risk. The combined form of the probability index is obtained in this study. Different accidents have different risk occurrence modes because accidents occur with different mechanisms. The indexes that can cause accidents are related and cannot be studied independently.

Considering the characteristics of subway construction, the occurrence modes of disastrous accidents can be concluded as follows.

#### 3.3.1. Design Indexes-Insensitive Mode

This mode considers the basic index as a risk source, together with improper construction management and operation, which leads to the occurrence of the risk. The design index does not often work in accidents. For example, the factors of shield axis risk control such as uniformity of formation, tunnel slope, and turning radius are mainly basic indexes; the construction indexes including the construction level, experience, and design indexes have a very insignificant relationship with the risk.

#### 3.3.2. Basic Index-Insensitive Mode

The risk of the occurrence of accidents in this mode has no relationship with the basic indexes; the occurrence is mainly caused by improper design and construction. For example, the deformation or damage to the base in the shield moving-out construction is mainly caused by the poor design safety coefficient and improper operation.

#### 3.3.3. Comprehensive Mode

The above two models involve only two types of indexes; however, most accidents occur because of the combined action of the attributes of the tunnel (strata, environment, tunnel diameter, buried depth, and others), design factors, and construction factors. These three kinds of indexes compose a relationship chain. The probability value changes when one of the indexes changes, such as the risk of collapse, during shield moving-out construction. 

The above three occurrence modes are the bases of risk assessment for an accident. An accident may involve one or more modes. The fault tree analysis method can be used to determine the accident occurrence mode.

The probability calculation model is obtained based on the above three modes.

(a) *Design Indexes-Insensitive Mode. *The probability index is affected by the basic and construction indexes. In view of the influence of the construction indexes on probability, the construction coefficient *C*_*k*_ is introduced:

(3) construction  coefficient  Ck  =construction  index  Cconstruction  standard  value  Cs,

where the construction standard value is a constant set in advance. It represents the construction level in general and does not reduce nor increase the disastrous accident probability.

The risk probability is given by

(4) P=B×CK=B×(CCS).

The value of the construction coefficient is about 1. If the value is greater than 1, the construction increases the risk of accidents and vice versa.

(b) *Basic Index-Insensitive Mode.* Similar to the design index-insensitive mode, the construction coefficient *C*_*k*_ and the construction standard value *C*_*s*_ are introduced. The calculation model is defined as

(5) P=D×CK=D×(CCS).

(c) *Comprehensive Mode.* The design coefficient *D*_*K*_ and construction coefficient *S*_*k*_ are introduced:

(6) design  coefficient  DK=  design  index  Ddesign  standard  value  Ds.

The calculation model of this mode is given by

(7) P=B×Dk×Ck.

### 3.4. Combining the Basic Indexes and Design Indexes

The simplest method to combine the basic and design indexes is to consider them separately and then establish the basic index vector. One has

(8) B=[B1,B2,B3,B4⋯Bm],

where *B*_i_ represents the *i*th basic index.

To establish the design index vector, the following equation is utilized:

(9) D=[D1,D2,D3,D4⋯Dn],

where *D*_*j*_ represents the *j*th design index.

The results of the calculation can be expressed as

(10) sum(B)×sum(D)(n×Ds).

**Figure 3:**
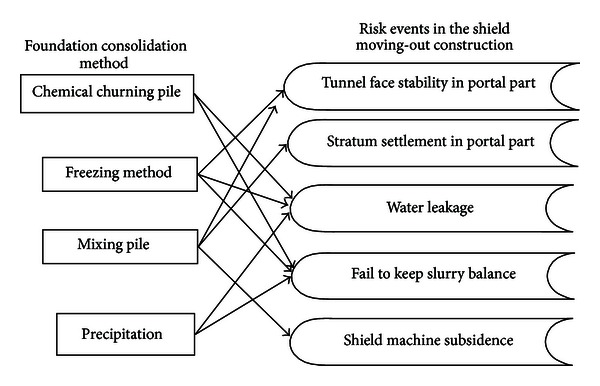
Relationship between formation reinforcement design and risk events.

In cases where the basic and design indexes are interrelated, this simple calculation method is feasible. However, in practical engineering, several design indexes may not affect all basic indexes. Considering the foundation consolidation risk in the shield moving-out construction as an example [18], different reinforcement designs have different effects. Several of these designs do not reduce the risk caused by several basic indexes, as shown in Figure 3. When the above calculation is used, the risk may appear to be lower or higher. Thus, the matrix *K*_*m*,*n*_ is introduced to solve this problem:

(11) K(i,j)={0,the  ith  basic  index  does  not  affect  the  jth  design  index,1,the  ith  basic  index  affects  the  jth  design  index,

where *i* = 1,2, 3,…, *m*, *j* = 1,2, 3,…, *n*.

The results of the calculation can be expressed as

(12) Bm×Km,n×(DDs).

### 3.5. Consequence Indexes

The indicators of the five kinds of losses can be obtained according to the literature [6]. The economic losses are utilized as an example, as shown in Table 2.

## 4. Risk Assessment Process

**Figure 4:**
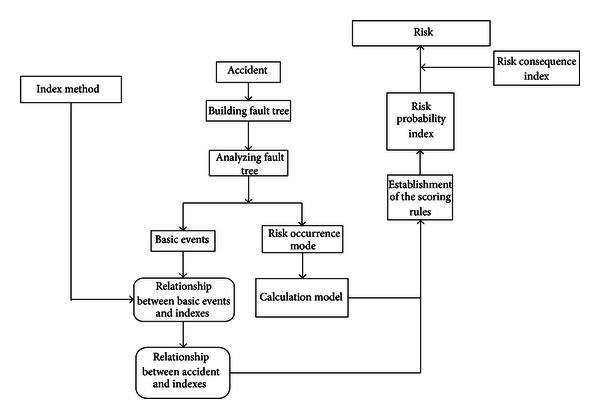
Risk assessment process diagram.

Disastrous accidents are risk assessment subjects. The initial steps are to identify the disastrous accidents that happen in subway engineering, to understand the risk occurrence mechanism, and to conduct risk assessment. The fault tree analysis method is a good way to recognize the accident occurrence mechanism. It can be used with other risk assessment methods. The basic events in an accident can be obtained by utilizing the fault tree method. Through the study of these basic events, the relationship between the accident and the indexes could be determined, the risk occurrence mode could be obtained, and the calculation model would be provided. The risk assessment process is shown in Figure 4.

## 5. Risk Acceptance Criteria

In accordance with the scoring rules and characteristics of the index method, the risk acceptance criteria are determined. The risk level is divided into four grades, namely, unacceptable, unwilling to accept, acceptable, and negligible, as shown in Table 3.

## 6. Case Study

The risk assessment conducted for the river-crossing tunnel construction of Nanjing subway line 10 was utilized as an example. The basic introduction of the tunnel is shown in Table 4.

### 6.1. Risk Assessment

The first step in risk assessment is risk identification, which aims to predict potential accidents and to determine the factors of these accidents. A longer tunnel may be divided into several sections according to the strata conditions before risk identification. The conditions of the tunnel's environment are affected by shield tunneling.

As the conditions along the subway line's route change, so does risk. Risk is not constant. Therefore, examining a long tunnel in shorter sections is more efficient. The risk evaluator must decide on a strategy to create sections to obtain an accurate risk value. Each section has its own risk assessment results. Breaking the subway line into many short sections increases the accuracy of the assessment for each section, but may result in higher costs of data collection, handling, and maintenance. Longer sections (fewer in number), on the other hand, may reduce data costs, but may also reduce accuracy because the average or worst case characteristics dominate if the conditions change within the section [9].

The considered attributes in sectioning or segmenting the tunnel include

- strata characteristics,
- environment conditions,
- buried depth.

**Figure 5:**
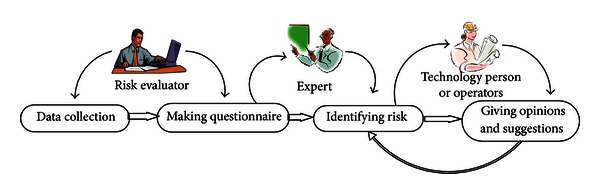
Risk identification process.

The next step is to identify the risk and risk factors. The risk identification process is shown in Figure 5. The main steps are collecting data, making the questionnaire, identifying the risk, and giving opinions and suggestions [19]. Three persons are involved in the risk identification process, namely, the risk evaluator, the expert, and the technology person or operators. The risk evaluators, who function as guides, collect data, make the questionnaires, and send the questionnaires to the experts for risk identification. The experts, as important participants, utilize their experience and finish the questionnaires. The technology person or operators, as the personnel in charge of practical engineering, give their opinions and suggestions on the identified risks, which are obtained by synthesizing the opinions of the experts. Based on the investigation, environment survey, shield selection, and preliminary design data, the risks in sections are identified and are shown in Table 5.

The fault tree and index methods are utilized to analyze and assess the accidents. Face instability, which is the most notable accident, is used as an example.

Based on the fault tree, the risk occurrence mode of face instability and the related basic, design, and construction indexes can be obtained. The results are shown in Table 6. 

### 6.2. Key Parameters in the Probability Index

**Figure 6:**
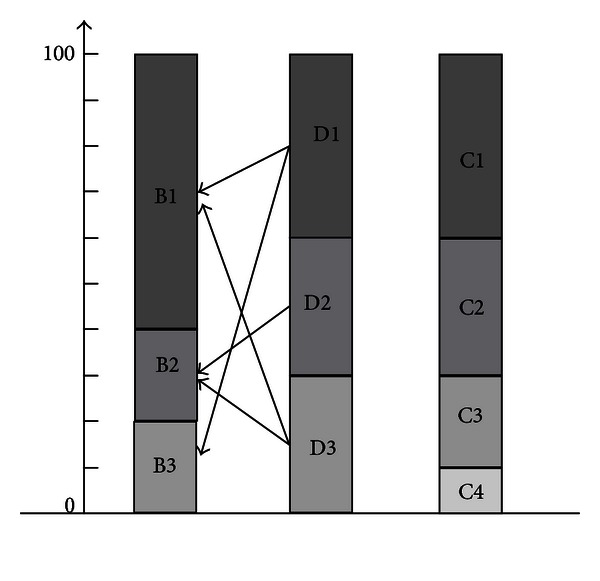
Relationship between the basic and design indexes.

(a) Standard value. The score scope, design standard value, and construction standard value are determined and shown in Table 7 in accordance with the risk calculation model.
(b) *K*_*m*,*n*_. Based on the construction experiences and the comprehensive scoring of the expert, the weights of each basic and design index and the relationship between the basic and design indexes are determined and shown in Figure 6.
 In Figure 6, the meaning of *B*_1_, *B*_2_, *B*_3_, *D*_1_, *D*_2_, *D*_3_, *D*_4_, *C*_1_, *C*_2_, *C*_3_, and *C*_4_ can be got from Table 6, and the length of the column represents the weights. The connecting line indicates the correlation between the design and basic indexes.
 Thus, we obtain
  (13) Km,n  is  [101011100].
(c) Weights. Obtaining the weights of the indexes is a very important task in risk assessment. The results of existing research and advanced study methods are fully utilized to obtain the weights. The factors that affect the limit support stress ratio, according to the results of existing research, mainly include the buried depth of the tunnel, friction angle and cohesion of the soil, and depth of the groundwater and river. However, no data provide the weights of these factors.

**Figure 7:**
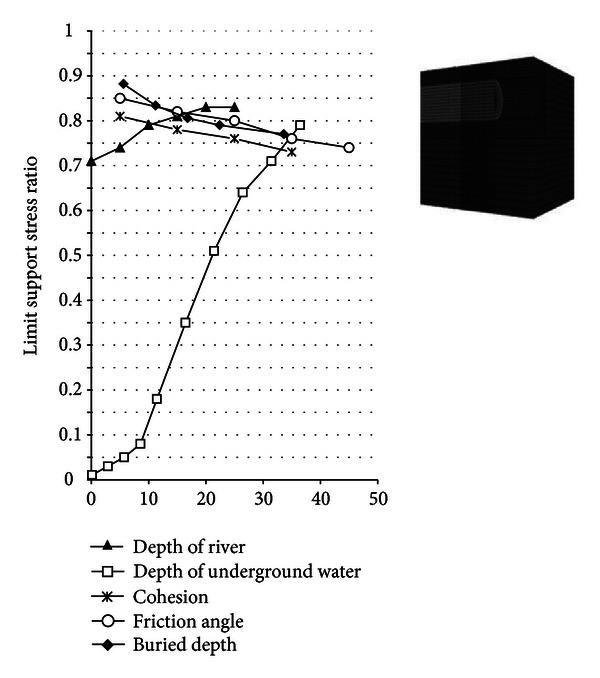
Simulation model and results.

The numerical simulation method is used to obtain the weights in this study. The simulation model is shown in Figure 7, which also contains the effects of the abovementioned factors on the limit support stress ratio.

From the numerical simulation results, increasing the buried depth, friction angle, and cohesion reduces the limit support stress ratio. However, increasing the depth of the underground water and the river increases the limit support stress ratio. Groundwater depth has the biggest impact on limit support stress ratio, followed by river depth, buried depth, cohesion, and friction angle. The weights of these factors are shown in Table 8.

### 6.3. Summary of Results and Analysis

Based on the three key parameters, risk assessment can be finalized after establishing a scoring rule. The accidents and the types of risks in each section must be provided.

**Figure 8:**
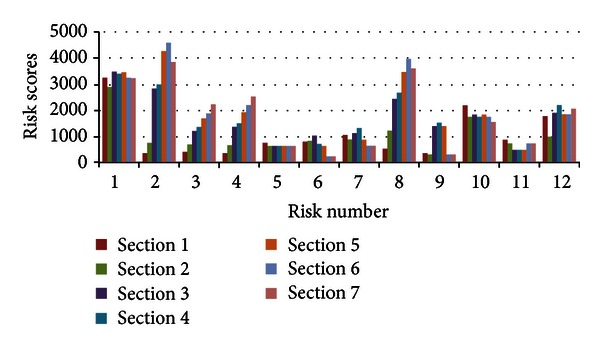
Accidents and risk scores in the different sections.

The accidents and risk scores in each section are shown in Figure 8. In the figure, the risks which are represented by risk number are shown in Table 5. These data are considered as the preliminary risk assessment results. They do not consider the influence of construction. The construction indexes are difficult to evaluate before the construction period because the construction index is more dependent on the attitude of the construction personnel (whether or not the construction personnel are active and conscientious). The results in Figure 8 reflect the influence of the basic and design indexes. The risk assessment is conducted to inform the construction personnel what risks they should pay more attention to and what measures to take. The risk events that have high scores are face instability, tool wear, and shield tail seal failure. We suggest that construction personnel pay special attention to these three risk events in the shield-driving process and prepare risk control measures.

**Figure 9:**
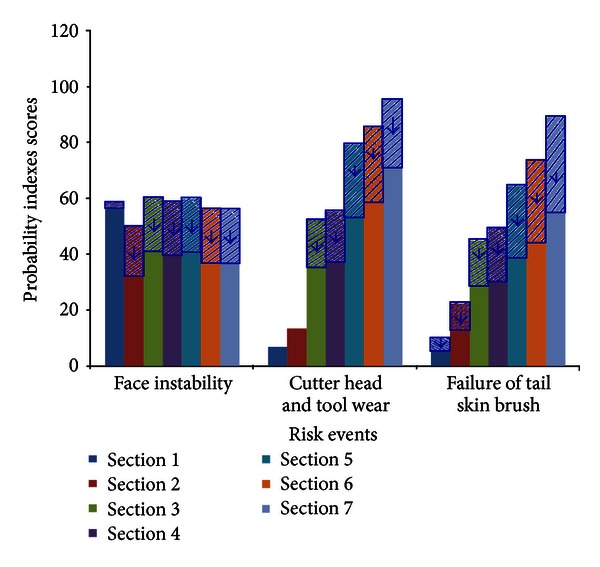
Risk probability index in the preliminary risk assessment and construction process.

During the construction period, the score of the construction indexes is obtained according to the measures that the construction personnel have taken and their attitude. The risk probability indexes in the preliminary risk assessment and the construction process (including the construction indexes) are shown in Figure 9. The shadow in Figure 9 represents the influence of the construction index. The arrowheads indicate the increase or decrease in the index score. An upward arrowhead means the score increases, whereas a downward arrowhead means the score is reduced. The probability index almost decreases because several measures have been taken to reduce the risk and more attention has been given to the risk events. However, for the face instability risk event in Section 1, the index increases because when tunneling begins, the operators of the shield machine need to adapt to the performance of the machine itself. They adjust and optimize the shield-tunneling parameters by monitoring the data until it achieves the optimum result. During this period, the construction increases the probability index. 

**Figure 10:**
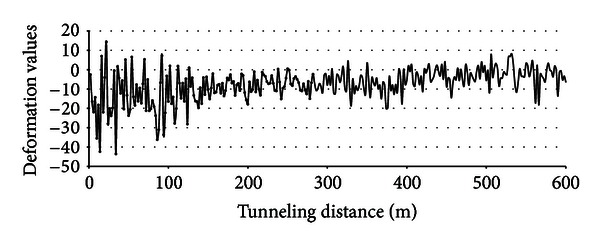
The frontal soil deformation values of the tunneling face.

Construction information is provided to verify the validity of this study's risk assessment model. Throughout the construction period, the face instability risk event does not happen. This finding is consistent with our risk assessment results. For the aforementioned risk event, the scores in most of the sections are around 40, which indicate that the probability is between “rarely” and “occasionally.” Only the first section has the score of about 60, which means that the event is close to “possible” but the probability is still “occasionally.” The frontal soil deformation of the tunneling face reflects the probability of the risk. The deformation values in the different sections are shown in Figure 10. At the initial point of 100 m, the range of the deformation values is between −30 mm and 10 mm. The deformation values at the other points are around −20 mm to 10 mm, and most of them are around −10 mm to 5 mm. The above analysis shows that the construction indexes affect the probability of the risk event. The changing trend is consistent with the result of the risk assessment.

**Figure 11:**
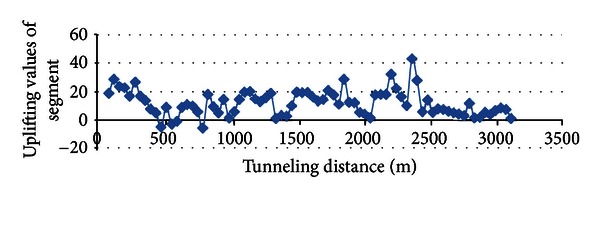
Values of segment uplifting.

The values of segment uplifting are also provided to verify the assessment model for the risk events, as shown in Figure 11. The segment-uplifting values are within the range of −10 mm to 30 mm, which is acceptable according to the code for the construction and acceptance of the shield-tunneling method [20].

Based on the monitoring data, risk assessment can be applied in subway construction. This assessment indicates which risks the construction personnel are not willing to accept, those they can accept, and those they ignore.

## 7. Conclusion and Discussion

The purpose of this paper is to establish a qualitative-quantitative risk assessment model. The risk events change as the external environment changes; thus, the probability of the risk events happening also changes. If the value of the change is not large, adjusting the risk score by using the qualitative analysis method becomes difficult. With regard to general qualitative risk assessment, this study's model should easily adapt to the dynamic changes of the risk. With regard to general quantitative risk assessment, this model does not need to obtain the “real” probability, a process which costs much money. The probability index can be obtained by substituting the data in the scoring model with the data obtained from an existing document or information. Our assessment model can be compared to a thermometer and the risk value to temperature. The thermometer indicates what clothes people should wear; similarly, through our assessment model, construction personnel are informed of the safety situation and also the risks involved. Thus, the construction personnel would know how to respond in case an accident occurs.

An improved index method is established to address the above purpose. Four kinds of indexes are considered in the improved method, namely, (1) basic, (2) design, (3) construction, and (4) consequence indexes. Indexes (1), (2), and (3) constitute the probability index, and the calculation model of which is provided based on three accident occurrence modes. Considering the correlation between the basic and the design indexes, the coefficient matrix   *K*_*m*,*n*_ is introduced. The basic index model is then finalized. Different risk events have different occurrence mechanisms and risk factors. Thus, no single formula can express the relationship between the risk index and the known parameters. In this paper, the risk assessment for the river-crossing tunnel construction of Nanjing subway line 10 was utilized as an example, and face instability as a risk event was discussed in detail.

This risk assessment model can be applied to the assessment of disastrous accidents in the design and construction stages. In the design stage, the results of risk assessment can be obtained without considering the construction indexes. The results of risk assessment can be an important reference in the selection of design schemes. The construction indexes are considered in the construction stage. Based on the current construction conditions, a dynamic risk assessment was carried out for dynamic security control during the period of construction.

This risk assessment model has been verified by the Nanjing subway engineering. When the risk occurrence mechanism is not completely clear, the analysis of the risk events still relies on previous engineering experience, which is very crucial for the reliability of the index model. John Hudson, a famous British geotechnical engineering expert, talked about the importance of “collective memory" in the International Top-Level Forum on Engineering Science and Technology Development Strategy-Safe Construction and Risk Management of Major Underground Engineering [21]. He suggested that previous engineering data and experience should be sorted to establish a big shared database. The authors think that the establishment of this database is very important for the ongoing development of risk assessment methods. 

## Figures and Tables

**Table 1:** Several typical subway construction accidents in China.

Time	City	Loss
2004-3-17	Guangzhou	1 death [10]
2007-3-28	Peking	6 deaths [11]
2007-2-5	Nanjing	The ground collapsed, and many residents were affected [12]
2008-11-15	Hangzhou	21 deaths [13]

**Table 2:** Indicators of economic losses.

Disaster grade	Disastrous	Very serious	Serious	Moderate	Slight
Economic loss (¥ × million)	>10	3–10	1–3	0.3–1	<0.3
Score	20	16	12	8	4

**Table 3:** Risk acceptance criteria.

Risk value	Risk grade	Risk acceptance level	Response principle
0–1600	IV	Negligible	Risk management can be implemented.
1600–3600	III	Acceptable	Risk management can be implemented, and risk management measures can be taken.
3600–4800	II	Unwilling to accept	Risk management should be implemented to reduce the risk, but the cost of reducing the risk must not be higher than the risk loss.
>4800	I	Unacceptable	Risk management measures must be taken to reduce the risk to grade II.

**Table 4:** Basic introduction of the tunnel.

Tunnel mileage	K11 + 251–K14 + 857
Tunnel design	Single tunnel, double track
Tunnel diameter (outer/inner)	11.2/10.2
Tunnel buried depth	5 m to 35 m
Shield type	Slurry shield
Stratum	silt layer, fine sand; 4-4e1 round gravel layer
Depth of river	0 m to 25.5 m
Permeability coefficient	5.43 × 10^−7^ to 8.87 × 10^−4^ cm · s

**Table 5:** Risks or accidents identified in Section 3.

No.	Risk/accident
1	Face instability
2	Cutter head and tool wear
3	Large size bearing breaking
4	Failure of bearing seal
5	Failure of hoisting jack
6	Mud cake
7	Clogging at the exit of slurry
8	Failure of tail skin brush
9	Failure of pushing axis control
10	leakage water at the segment
11	Failure of segment erection
12	Segment uplift
13	Jammed grouting pipe
14	Bad grouting effect

**Table 6:** Risk occurrence mode of face instability and the related indexes.

Occurrence mode	Comprehensive mode
Related indexes	
Basic indexes (no.)	Affects the limit support stress ratio (B1) Change of strata (B2) Overbreak of the affected strata (B3)
Design indexes (no.)	Shield-excavating equipment design (D1) Shield-pushing equipment design (D2) Strata adaptability of the shield (D3)
Construction indexes (no.)	Level of the construction technology (C1) Construction period (C2) Construction environment (C3) Control measures (C4)

The limit support stress ratio is the ratio of the value of limit support stress to original lateral geostress, where the limit support stress refers to the minimum stress that can support face stability.

**Table 7:** Several key parameters.

Indexes	Score scope	Notes
Basic index Design index Construction index *D*_*k*_ *S*_*k*_	0–100 0–100 >0 50 50	If the score is lower, the probability index is also lower. The construction index is unlimited, which shows that even though the basic index is not large, the possibility of an accident happening would still be high in poor construction conditions.

**Table 8:** Weights of the factors that affect the limit support stress ratio.

Factor	Weight
River depth	0.13
Buried depth	0.11
Friction angle	0.05
Cohesion	0.07
Depth of underground water	0.64
